# Is proximity to a food retail store associated with diet and BMI in Glasgow, Scotland?

**DOI:** 10.1186/1471-2458-11-464

**Published:** 2011-06-10

**Authors:** Laura Macdonald, Anne Ellaway, Kylie Ball, Sally Macintyre

**Affiliations:** 1 MRC/CSO Social & Public Health Sciences Unit, 4 Lilybank Gardens, Glasgow, G12 8RZ, Scotland, UK; 2 School of Exercise and Nutrition Sciences, Deakin University, 221 Burwood Highway, Burwood VIC 3125 Australia

## Abstract

**Background:**

Access to healthy food is often seen as a potentially important contributor to diet. Policy documents in many countries suggest that variations in access contribute to inequalities in diet and in health. Some studies, mostly in the USA, have found that proximity to food stores is associated with dietary patterns, body weight and socio-economic differences in diet and obesity, whilst others have found no such relationships. We aim to investigate whether proximity to food retail stores is associated with dietary patterns or Body Mass Index in Glasgow, a large city in the UK.

**Methods:**

We mapped data from a 'Health and Well-Being Survey' (n = 991), and a list of food stores (n = 741) in Glasgow City, using ArcGIS, and undertook network analysis to find the distance from respondents' home addresses to the nearest fruit and vegetable store, small general store, and supermarket.

**Results:**

We found few statistically significant associations between proximity to food retail outlets and diet or obesity, for unadjusted or adjusted models, or when stratifying by gender, car ownership or employment.

**Conclusions:**

The findings suggest that in urban settings in the UK the distribution of retail food stores may not be a major influence on diet and weight, possibly because most urban residents have reasonable access to food stores.

## Background

Access to healthy food is often seen as a potentially important contributor to a healthy diet. Policy documents in many countries suggest that lack of such access in certain areas is one explanation for inequalities in diet and in health [1,2]. A growing number of studies have explored links between the local food retail environment, and dietary habits or overweight/obesity. Recent reviews report inconclusive results [3-7]. For example, a number of studies based in regions of the USA found that better supermarket access was associated with increased fruit and vegetable intake [8], and dietary quality [9,10], and reduced levels of overweight/obesity [11-17]. Higher intakes of fruit and vegetables were seen amongst those with better access to large, non-chain grocery stores [18], and smaller food stores [19,20], while a shorter distance to fruit and vegetable stores was associated with healthier body mass index (BMI) [16]. On the other hand, in some studies, better access to supermarkets [21,22], convenience stores [14,15,17], and small grocery stores [13,22] was associated with increased levels of overweight/obesity, while no significant links were found between supermarket proximity and fruit and vegetable intake [18], or between small grocers, convenience stores, and BMI [11].

Evidence from outside the USA about associations between neighbourhood environments and diet or obesity is also mixed. A New Zealand study found no association between fruit and vegetable intakes and better access to food stores, but living near a convenience store was negatively associated with vegetable intake [23], while a study in Australia found that supermarket density was not associated with fruit and vegetable intake [24]. In Japan there were no greater intakes of fruit and vegetables with better access to fruit and vegetable stores, grocery stores or supermarkets [25]. Several UK studies have found no links between supermarket proximity and fruit and vegetable intake [26] or levels of obesity [27]; however a recent study across the Republic of Ireland did find a link between nearness to a supermarket and better diet [28].

Given this observed variation across studies, there is a need for further investigation of the associations between retail food environments and both diet and body weight [29]. This is particularly important in countries outside of the USA, in the light of the relative dearth of international data, and evidence of systematic differences in food store distribution patterns between the USA and other countries. We have previously shown that, unlike in many cities in the USA, the distribution of various types of food retailer in Glasgow does not disadvantage poorer socio-economic groups [30-32]. In this paper we investigate whether proximity to various types of food stores is associated with dietary patterns and BMI. We also explore these relationships separately by gender as a number of studies have found gender differences in the relationship between the neighbourhood environment and health and health behaviours [22,33-36], and we have previously shown in Glasgow that 87% of female respondents, compared to 30% of males, reported doing most of the food shopping for their household [37]. We also examine these relationships separately by household car access and employment status, since these might be hypothesised to influence food purchasing patterns, and we might expect the association between neighbourhood food environments and diet/obesity to be stronger amongst those who spend more time in their neighbourhoods.

## Methods

We capitalised on two existing datasets relating to Glasgow City, Scotland: a 'Health and Well-Being Survey' (HWB, 2002), conducted in 2002 by the Greater Glasgow Health Board (GGHB), and a list of food retailers in 2007 held as part of the Glasgow City Council Public Register of Food Premises.

The HWB sample was stratified proportionately by local authority and deprivation category (DEPCAT), with addresses selected randomly. Over two thirds (67%) of individuals contacted took part in the study which led to 1802 face-to-face interviews with adults in the GGHB in 2002. Data were gathered on individuals' socio-demographic characteristics, health and health behaviours. Data were weighted to ensure that they were representative of the adult population in this area [38]. In this paper we used only Glasgow City respondents (N = 1149) since the list of food stores was for Glasgow City. We used two measures of diet: daily consumption of fruit and vegetables, and daily consumption of high-fat snacks, as these data were available within the survey and because these two key indicators of diet have been associated with obesity risk [39,40]. For fruit and vegetable consumption we created a variable of eating five or more portions of fruit and vegetables daily versus less than this, based on two questions (*'On average, how many portions of fruit do you eat each day?'*, and *'On average how many portions of vegetables or salad (not counting potatoes) do you eat each day?'*). The high-fat snack consumption variable was based on responses to the question *'How often PER DAY do you usually eat items such as cakes, pastries, chocolate, biscuits and crisps*?', which we grouped into none or one versus at least two (we also explored a grouping of none versus one or more). The former categorisation (none or one versus at least two) was used by the GGHB within the HWB report [38]. BMI was calculated from self-reported height and weight measurements, from which we constructed a three-category variable ('healthy weight' 18.5-24.99, 'overweight' 25-29.99, 'obese' 30 and over) and also a two-category variable with a threshold of <25 v 25+. Respondents who were underweight (BMI less than 18.5, N = 37) were excluded from the analysis. We used measures of household car ownership (household had access to one or more cars, or none) and of employment status (as a dichotomous variable, with those in fulltime employment, training or education categorised as 'employed' and those who were unemployed, permanently sick/disabled, retired or homemakers categorised as 'not employed'). Socio-economic position was represented by an occupationally based 'Socio-Economic Group' classification, which had been grouped into three categories ('A/B/C1', 'C2', 'D/E'). These socio-demographic variables were included since they might influence ease of access and proximity to food stores, and the amount of time spent in the local neighbourhood.

The list of food retailers (n = 741) was held by the Council for licensing, inspection and planning purposes, and included all premises that fall under the 1995 Food Safety and Hygiene guidelines [41]. We used the Council defined categories, 'supermarket' (which included large chain supermarkets and superstores, selling a wide range of food products n = 68), 'general store' (which included smaller independent food stores and chain outlets, selling a smaller range of food products n = 637), and fruit and vegetable stores (n = 36). We excluded other categories on the Council list such as delicatessens, butchers, fishmongers etc.

We obtained street maps (including point addresses) from the UK Ordnance Survey [42], and used ArcGIS version 9.1 software to geocode respondents and food retailers by their unit postcodes. We carried out network analysis (i.e. found the shortest path between two locations on a road network) to find the network distance in metres from each respondent to the nearest supermarket, general store and greengrocers.

Network distance to the nearest general store was dichotomised with a threshold of 500 metres, while network distance to the nearest fruit and vegetable store and nearest supermarket was dichotomised by 1000 metres. We chose 500 metres for the nearest general store to ensure the stores were local to the respondents, and because initial inspection of the data showed that application of a larger threshold for this variable resulted in too little variability (i.e. 98% of participants lived within 1000 metres of a general store). The 1000 metre threshold for the other stores represented an approximately 15 minute walk for adults in an urban area and this has been demonstrated as an appropriate distance in previous literature [28,43-47]. This threshold also produced a distribution suitable for analysis.

Logistic regression was used to explore associations between snack intake and distance to the nearest general store or supermarket; and between fruit and vegetable consumption and distance to the nearest fruit and vegetable store or supermarket. Multinomial regression was used to examine whether proximity to food retailers was associated with BMI category (using the three-category BMI outcome measure and also the two-fold variable). In addition we used GLM to explore whether proximity (as a continuous variable) was associated with diet and BMI as continuous outcome variables. We examined, firstly, unadjusted odds ratios for diet and BMI; secondly odds ratios adjusted for age, gender, and socio-economic position; and thirdly odds ratios adjusted for age, gender, socio-economic position, car ownership and employment status. We also formally explored various interactions within the models (e.g. age and gender; gender and employment status; gender and car ownership). We then conducted a stratified analysis by gender (controlling for age and socio-economic position), household car access (controlling for age, gender and socio-economic position), and employment status (controlling for age, gender and socio-economic position). Within the HWB survey 991 respondents had no missing values for any of the variables to be included in the analysis (see table 1).

**Table 1:** GGHB 'Health and Well-Being Survey 2002' Respondents (n = 991)

		N	%
Age category	16-24 years old	110	11.1
	25-34 years old	157	15.8
	35-44 years old	165	16.6
	45-54 years old	130	13.1
	55-64 years old	120	12.1
	65-74 years old	170	17.2
	75+ years old	139	14.0
Gender	Male	394	39.8
	Female	597	60.2
Socio-Economic Position	A, B, C1	346	34.9
	C2	225	22.7
	D, E	420	42.4
Household owns a car	Yes	390	39.4
	No	601	60.6
Employment status	Employed, full time student	343	34.6
	Unemployed, retired, homemaker	648	65.4
Body Mass Index	18.5-24.99	559	56.4
	25-29.9	310	31.3
	30+	122	12.3
Fruit/vegetable consumption	Less than 5 portions every day	680	68.6
	At least five portions every day	311	31.4
High-fat snack consumption	2 or more high-fat snacks daily	309	31.2
	1 or less high-fat snack daily	682	68.8
Access to...			
General store (within 500 m)		723	73.0
Fruit/vegetable retailer (within 1000 m)		313	31.6
Supermarket (within 1000 m)		458	46.2

## Results

Almost three quarters (73%) of the HWB sample lived within 500 metres (m) of a general store, 32% within 1000 m of a fruit and vegetable store, and 46% within 1000 m of a supermarket.

We found few statistically significant associations between proximity to food outlets and diet or BMI. There was little difference in fruit and vegetable consumption by proximity to fruit and vegetable stores, and although the odds of obesity appeared lower in those living within 1000 m of a fruit and vegetable store this difference was not statistically significant. Odds of eating more than one high-fat snack daily, eating less than five portions of fruit and vegetables daily, and obesity were higher among those within 1000 m of a supermarket but again these results were non-significant. The only association statistically significant at the 5% level was that between obesity and proximity to general stores after adjustment for age, gender and socio-economic position (see table 2) and after adjustment for age, gender, socio-economic position, car ownership and employment status (see table 3). Results were broadly similar when distance and dietary intake were used as continuous, rather than categorical, variables in the analyses, and when BMI was included as a twofold category (data not shown). Results were similar for the odds of daily consumption of any snacks (data not shown). We found no significant interactions between age and gender, or between gender and employment status, or gender and car ownership (data not shown).

**Table 2:** Odds of meeting dietary recommendations, being overweight/obese, by store proximity (controls - age, gender, SEP)

	N	General store within 500 m Odds Ratio (95% CI)	Fruit & vegetable store within 1000 m Odds Ratio (95% CI)	Supermarket within 1000 m Odds Ratio (95% CI)
**ALL RESPONDENTS**	991			
				
***Diet***				
*Eats high-fat snacks >1 daily*				
*no*	682	1.00		1.00
*yes*	309	1.24 (0.90-1.70), p = 0.190		1.13 (0.86-1.49), p = 0.385
*Eats fruit & vegetables, >5 daily*				
yes	457		1.00	1.00
no	534		0.97 (0.71-1.31), p = 0.826	1.28 (0.97-1.71), p = 0.086
***BMI***				
*18.5-24.99*	559	1.00	1.00	1.00
*25-29.99*	310	1.13 (0.82-1.56), p = 0.444	0.99 (0.73-1.36), p = 0.973	1.04 (0.78-1.39), p = 0.786
*30+*	122	*1.74 (1.06-2.85), p = 0.028*	0.79 (0.50-1.26), p = 0.325	1.35 (0.89-2.03), p = 0.153

**Table 3:** Odds of meeting dietary recommendations, being overweight/obese, by store proximity (controls - age, gender, SEP, car ownership, employment)

	N	General store within 500 m Odds Ratio (95% CI)	Fruit & vegetable store within 1000 m Odds Ratio (95% CI)	Supermarket within 1000 m Odds Ratio (95% CI)
**ALL RESPONDENTS**	991			
				
***Diet***				
*Eats high-fat snacks >1 daily*				
no	682	1.00		1.00
yes	309	1.22 (0.88-1.68), p = 0.229		1.12 (0.85-1.48), p = 0.413
*Eats fruit & vegetables, >5 daily*				
yes	457		1.00	1.00
no	534		0.94 (0.69-1.28). p = 0.702	1.20 (0.92-1.56), p = 0.189
***BMI***				
18.5-24.99	559	1.00	1.00	1.00
25-29.99	310	1.15 (0.83-1.58), p = 0.405	0.99 (0.73-1.36), p = 0.994	1.05 (0.78-1.40), p = 0.755
30+	122	*1.80 (1.09-2.96), p = 0.021*	0.81 (0.51-1.28), p = 0.362	1.37 (0.91-2.07), p = 0.136

In the stratified analysis, as with the analysis of the total sample, there were few statistically significant associations (see table 4). There were no significant findings for females and non-car owners. Males were significantly less likely to eat five portions of fruit and vegetables daily if they lived within 1000 m of a supermarket (p <0.01). Car owners had significantly greater odds of snacking (p <0.05), and of being obese (p <0.05), if they lived within 500 m of a general store. Employed respondents were more likely to be obese when living within 500 m of a general store (p <0.05), and less likely to be obese when living within 1000 m of a fruit and vegetable store (p <0.05), while the unemployed were more likely to be obese when living closer to a supermarket (p <0.05).

**Table 4:** Odds of meeting dietary recommendations, being overweight/obese, by store proximity, stratified by gender, car ownership, employment

	N	General store within 500 m Odds Ratio (95% CI)	Fruit & vegetable store within 1000 m Odds Ratio (95% CI)	Supermarket within 1000 m Odds Ratio (95% CI)
***MALES***	394			
***Diet***				
*Eats high-fat snacks >1 daily*				
no	273	1.00		1.00
yes	121	1.35 (0.83-2.19), p = 0.232		1.14 (0.73-1.78), p = 0.567
*Eats fruit & vegetables >5 daily*				
yes	192		1.00	1.00
no	202		0.85 (0.53-1.38), p = 0.517	*1.81 (1.16-2.82), p = 0.009*
***BMI***				
18.5-24.99	220	1.00	1.00	1.00
25-29.99	142	1.21 (0.76-1.93), p = 0.414	0.98 (0.61-1.59), p = 0.940	0.80 (0.52-1.24), p = 0.322
30+	32	1.86 (0.77-4.46), p = 0.166	0.99 (0.41-2.44), p = 0.997	1.24 (0.57-2.70), p = 0.580

**FEMALES**	597			
***Diet***				
*Eats high-fat snacks >1 daily*				
no	409	1.00		1.00
yes	188	1.21 (0.79-1.85), p = 0.378		1.14 (0.80-1.64), p = 0.462
*Eats fruit & vegetables >5 daily*				
yes	265		1.00	1.00
no	332		1.02 (0.68-1.53), p = 0.920	1.01 (0.69-1.48), p = 0.948
***BMI***				
18.5-24.99	339	1.00	1.00	1.00
25-29.99	168	1.02 (0.65-1.60), p = 0.926	0.98 (0.65-1.49), p = 0.939	1.28 (0.86-1.89), p = 0.220
30+	90	1.81 (0.99-3.34), p = 0.055	0.81 (0.47-1.39), p = 0.437	1.47 (0.90-2.41), p = 0.122

***CAR OWNER***	390			
***Diet***				
*Eats high-fat snacks >1 daily*				
no	283	1.00		1.00
yes	107	*1.76 (1.04-2.99), p = 0.035*		0.80 (0.50-1.28), p = 0.347
*Eats fruit & vegetables >5 daily*				
yes	225		1.00	1.00
no	165		0.94 (0.60-1.48), p = 0.802	1.52 (0.99-2.32), p = 0.051
***BMI***				
18.5-24.99	221	1.00	1.00	1.00
25-29.99	120	1.48 (0.89-2.46), p = 0.127	0.71 (0.42-1.19), p = 0.188	1.01 (0.63-1.62), p = 0.959
30+	49	*2.61 (1.19-5.75), p = 0.017*	0.79 (0.36-1.72), p = 0.545	1.31 (0.66-2.60), p = 0.449
				*continued onto next page*

**NO CAR**	601			
***Diet***				
*Eats high-fat snacks >1 daily*				
No	399	1.00		1.00
Yes	202	0.96 (0.64-1.45), p = 0.853		1.32 (0.93-1.89), p = 0.118
*Eats fruit & vegetables >5 daily*				
Yes	232		1.00	1.00
No	369		0.91 (0.60-1.38), p = 0.647	1.06 (0.71-1.58), p = 0.765
***BMI***				
18.5-24.99	338	1.00	1.00	1.00
25-29.99	190	0.97 (0.63-1.48), p = 0.881	1.21 (0.81-1.79), p = 0.350	1.09 (0.75-1.58), p = 0.648
30+	73	1.40 (0.72-2.69), p = 0.320	0.88 (0.49-1.56), p = 0.658	1.38 (0.81-2.34). p = 0.232

***EMPLOYED***	343			
***Diet***				
*Eats high-fat snacks >1 daily*				
no	242	1.00		1.00
yes	101	1.38 (0.78-2.42), p = 0.266		0.84 (0.51-1.38), p = 0.488
*Eats fruit & vegetables >5 daily*				
yes	197		1.00	1.00
no	146		0.87 (0.54-1.39), p = 0.553	1.30 (0.83-2.05), p = 0.256
***BMI***				
18.5-24.99	214	1.00	1.00	1.00
25-29.99	96	1.02 (0.59-1.77), p = 0.933	0.90 (0.53-1.53), p = 0.698	0.81 (0.49-1.35), p = 0.424
30+	33	*3.07 (1.08-8.77), p = 0.036*	*0.35 (0.13-0.99), p = 0.048*	0.90 (0.40-2.01), p = 0.787

***NOT EMPLOYED***	648			
***Diet***				
*Eats high-fat snacks >1 daily*				
no	440	1.00		1.00
yes	208	1.14 (0.77-1.68), p = 0.517		1.28 (0.91-1.80), p = 0.150
*Eats fruit & vegetables >5 daily*				
yes	260		1.00	1.00
no	388		0.91 (0.68-1.21), p = 0.519	1.18 (0.90-1.55). p = 0.226
***BMI***				
18.5-24.99	345	1.00	1.00	1.00
25-29.99	214	1.17 (0.78-1.74), p = 0.453	1.03 (0.70-1.52), p = 0.876	1.16 (0.81-1.65), p = 0.423
30+	89	1.45 (0.82-2.57), p = 0.198	1.04 (0.61-1.77), p = 0.876	*1.64 (1.01-2.67), p = 0.045*

## Discussion

There were few clear or significant associations between proximity to food outlets and diet or BMI, either within the overall sample or within subgroups (e.g. men or women, households without a car(s) or households with a car(s), non-employed or employed). We did find an association between proximity to a supermarket and not eating fruit and vegetables which is puzzling, given that we controlled for age, gender and socio-economic positions, all of which may be associated with fruit and vegetable consumption. It is possible that proximity to a supermarket is a marker of proximity to a range of destinations selling energy dense foods. Contrary to expectations, associations were stronger among men, car owners and those in employment, a finding which is also puzzling since most hypotheses are that females, those out of the labour market and those without access to a car are more likely to be sensitive to exposures in their immediate residential environment.

Methodological limitations include the use of self-reported height and weight to calculate BMI, which may underestimate the true prevalence of overweight and obesity. For example, the proportions of respondents overweight and obese in the Health and Well-being survey (31.3% and 12.3% respectively) are considerably lower than the proportions in the Glasgow area obtained by direct measurements in the Scottish Health Survey 2003 (37.0% and 23.4%) [38]. However, we do not know of any evidence to suggest systematic biases in underreporting by proximity to food store, gender or deprivation which would affect our analysis.

The list of food stores was for 2007, five years after the HWB data collection. However a recent comparison of Council held list of stores available in 1997 and 2007 showed considerable consistency (even when a food store had closed, often another similar type was occupying the premises) (Cummins, personal communication), and verification on the ground in 2007 showed 88% of the 2007 list to be present and trading under the same name [48].

We recognise that residential proximity may not be the most important factor influencing food purchasing; however we did not have access to information on where people shop (they might use stores near their place of work or study, or near their child's school). A Canadian study highlighted the importance of investigating environmental influences outwith the local home neighbourhood; travel survey data and retail food store locations were used to create a measure of the types of stores to which participants were exposed while carrying out their daily activities [49]. It was found that people's 'foodscapes' differ whether one considers food environment exposures around the home or further afield and that these 'foodscapes' also differ by age and by income. In an earlier study conducted in Glasgow, we found that people with lower incomes were more likely to shop for basic foodstuffs in local shops than in shops further afield [37].

In this paper we did not cover accessibility, affordability, or quality of healthy food items within local shops, which might be more important for diet than proximity to a food store. A survey of all food shops within 9 Scottish areas found that healthy food accessibility was determined by the types of stores in an area and by the stores' stocking policy [50]. There was a consistently high level of availability in large and medium general stores [51], while availability in small general food stores depended on urbanicity or rurality [52]. In urban areas people who depended on small food stores had limited access to healthy foods, and their food would cost more, than if they used larger stores in their area [50]. We are pursuing similar issues using data on price and availability of a basket of foods in food stores in Glasgow, and their relationship to deprivation, and this will be reported in a separate paper.

Our results differed to those of the majority of USA studies; we did not observe that living closer to a supermarket was associated with increased fruit and vegetable intake, or with reduced levels of overweight/obesity, and did not find a link between proximity to a fruit and vegetable store and healthier BMI ([8,11-16]). In line with a study in Japan [25] we found that fruit and vegetable intake was not higher with better access to fruit and vegetable stores or supermarkets, and similarly in line with another UK study we found no link between supermarket proximity and respondents' levels of obesity [27].

## Conclusion

Despite its limitations we believe this study has contributed to the limited UK literature on the potential effect of local food retail upon diet and levels of overweight/obesity, and has extended existing studies by looking at various food types and food stores. Our overall finding of a lack of consistent associations highlights the importance of national context, i.e. that findings should not be extrapolated from, for example, urban USA to other countries with different patterns of urban dwelling and retail markets [32,53]. Our previous findings that supermarkets are more likely to be located in poorer areas of Glasgow further illustrate the importance of not overgeneralising from one society to another [30]. One major feature of contemporary British cities may be that there is a sufficient spread and density of food retail stores such that no population groups are significantly disadvantaged in access to food (e.g. 98% of HWB respondents lived within 1000 m of a general store, and 46% within 1000 m of a supermarket). In order to detect environmental determinants of food purchasing, diet and weight one might need to seek an environment with considerably more variation in food access than is observed in Glasgow.

Directions for further research might include exploring the healthiness and quality of what food retail outlets actually stock and promote in particular areas. It would also be useful to have access to actual consumer shopping behaviour, to ascertain what food people purchase, what shops and other food sources they use, and to understand the factors which influence food shopping. More sensitive comprehensive indicators of environmental exposures, such as 'foodscapes' or 'activity spaces' [49], would also be valuable.

## Competing interests

The authors declare that they have no competing interests.

## Authors' contributions

LM did the mapping, data analysis and the literature review. AE, KB and SM contributed to the conception of the paper and discussion of the analysis. All authors contributed to successive drafts of the paper. All authors read and approved the final draft. LM is guarantor.

## Pre-publication history

The pre-publication history for this paper can be accessed here:

http://www.biomedcentral.com/1471-2458/11/464/prepub
